# A Pilot Fuzzy System with Virtual Reality for Mild Cognitive Impairment (MCI) Assessment

**DOI:** 10.3390/healthcare11182503

**Published:** 2023-09-09

**Authors:** Cheng-Li Liu, Che-Jen Chuang, Chin-Mei Chou

**Affiliations:** 1 Department of Mechanical and Industrial Engineering, Vanung University, Taoyuan 320313, Taiwan; 2 Department of Airline and Transport Service Management, Vanung University, Taoyuan 320313, Taiwan; tiny@vnu.edu.tw; 3 Department of Industrial Engineering and Management, Yuan Ze University, Taoyuan 320315, Taiwan; kinmei@saturn.yzu.edu.tw

**Keywords:** mild cognitive impairment, Alzheimer’s disease, virtual reality, fuzzy logic control, screening tool

## Abstract

Mild cognitive impairment (MCI) is when brain function declines. MCI is the gray area transitioning from normal aging to the AD stage. Currently, the majority of early MCI diagnoses are processed through comprehensive neuropsychological tests. These tests may take the form of interviews, paper-and-pencil tests, or computer-based tests. There may be resistance from the subject if he/she has to undergo many screening tests simultaneously for multiple evaluation information, resulting in execution difficulty. The objectives of this study are to use 3D virtual reality to create an entertaining test scenario integrating the Mini-Cog, SPMSQ, MMSE, SLUMS, CDR, and CASI for middle-aged to older adults, furthermore, to employ fuzzy logic control (FLC) technology to develop a “MCI assessment system” for obtaining some pilot information for MCI assessment. There were 24 middle-aged to older adults aged from 50 to 65 years who participated in the evaluation experiment. The results showed that the MCI assessment system developed in this study is highly correlated with the traditional screening tests, including the Mini-Cog, SPMSQ, MMSE, SLUMS, and CASI. The assessment system can provide an integrated reference score for clinic workers in making judgments. In addition, the distribution of the System Usability Scale (SUS) evaluation scores for the MCI assessment system revealed that 87.5% were grade C (good to use) or above and 29.2% were grade B (extremely good to use) or above. The assessment system received positive feedback from the subjects.

## 1. Introduction

Mild cognitive impairment (MCI) is an early stage of memory defects, cognitive impairment, and difficulties in language or learning in individuals; but, it is different from dementia [1]. Regarding cognitive impairment, the National Institute of Mental Health was the first to propose the term “age-associated memory impairment” in 1986 [2]. Following that, the International Psychogeriatric Association proposed the term “age-associated cognitive decline” in 1994 to describe mild cognitive impairment (MCI) in patients who do not meet the criteria for dementia and whose activities of daily living are not affected [3]. The term “MCI” first appeared in the Global Deterioration Scale developed by Reisberg et al. (1982) to identify the tertiary symptoms of MCI in cognitive decline [4]. Patients with these symptoms have decreased processing capacity when faced with complex tasks or environments; but, simple activities of daily living are unaffected. Subsequently, Petersen et al. (2009) defined patients with classical MCI as those who have a memory defect, have poorer memory than people their age, do not have other cognitive problems, and do not meet the medical criteria for suspected Alzheimer’s disease (AD) [5]. They also proposed a related definition:

- Patients who have a subjective complaint of memory decline that family members or an observer can confirm;
- Patients with a poorer memory than those of the same age and education level;
- Patients with no defects in most normal cognitive functions;
- Patients who have basic activities of daily living;
- Patients who do not have dementia.

Although MCI patients are not affected regarding activities of daily living, some patients may exhibit persistent amnesia and difficulty calculating and organizing such symptoms as dementia.

The Diagnostic and Statistical Manual of Mental Disorders, 5th edition (DSM-5), published by the American Psychiatric Association in 2013, classified dementia into six major categories, namely, neurodegenerative dementia, vascular dementia, dementia caused by general medical conditions (AIDS, Huntington’s disease), dementia induced by substances (anticonvulsants, organophosphate insecticides), dementia with multiple etiologies, and dementia due to other factors [6]. Alzheimer’s disease (AD), frontotemporal dementia, and dementia with Lewy bodies account for 50%–75%, 5%–10%, and <5% of neurodegenerative dementia, respectively [7]. The earliest signs of AD, which accounts for most patients with neurodegenerative dementia, are memory decline and difficulties in identifying time, place, people, and objects. AD is a progressive and irreversible neurodegenerative disease [8]. The earliest apparent symptom of AD is forgetfulness. However, family members often dismiss progressive memory decline as a normal aging phenomenon, resulting in delayed treatment and worsening patients’ conditions [9]. There is currently no protocol for treating or slowing the progression of AD. However, many new treatments are in different stages of clinical trials. Various studies have found that if AD is detected early, patients can reduce their disease risk by exercising regularly, quitting smoking, avoiding alcohol consumption, controlling weight, consuming a healthy diet, and maintaining healthy blood pressure, cholesterol, and blood glucose levels. Although age is the most important known risk factor for AD, the disease does not only affect the elderly; in fact, 9% of AD cases occur at a younger age (i.e., symptoms occur before the age of 65 years). Therefore, early detection, proper treatment, and care can reduce the impact on family members and society [10].

What is the relationship between MCI and AD? Some researchers found that MCI is the gray area transitioning from normal aging to the AD stage [10]. Epidemiological studies have shown that MCI may be an underlying symptom of AD [11,12]. According to Peterson et al. (2014), the mean prevalence of MCI among the elderly over 70 years old was 14%–18% [13]. If these MCI patients have a history of memory deficits, there is a 10%–15% chance of developing AD in the future. In Taiwan, approximately 33% of patients with MCI identified through community screening will develop AD within 5 years and 10%–15% of cases will progress to AD every year, compared to 1%–2% in the same age group. MCI can be considered a high-risk AD factor [13]. Wang et al. (2006) found that AD’s most common pathological phenomenon is hippocampal atrophy, associated with amnestic MCI [14]. If patients with MCI have significant hippocampal atrophy, their risk of developing AD is exceptionally high. Therefore, screening for cognitive decline in the primary care setting with one or two of the neuropsychological tests is important and it should be confirmed in a memory clinic or specialized consultancy.

Currently, the majority of early MCI diagnoses are processed through comprehensive neuropsychological tests. These tests may take the form of interviews, paper-and-pencil tests, or computer-based tests. The tests are designed to assess memory, reasoning, attention, language, visual functions, motor functions, and social functions. However, each assessment tool has its diagnostic focus and many tests are often required to obtain a complete diagnosis. The common early screening tools used in clinical practice include Mini-Cog and the Short Portable Mental Status Questionnaire (SPMSQ). Neuropsychological tests include the Mini-mental Status Examination (MMSE), Saint Louis University Mental Status Examination (SLUMS), Clinical Dementia Rating (CDR), and cognitive abilities screening instrument (CASI). The contents and scoring methods of these screening tools and assessment scales are described below.

- Mini-Cog

The Mini-Cog was developed by Borson in 2000 to detect cognitive impairment in 3 min [15]. It is a short test used to identify people with poor memory and thinking abilities and requires the least amount of language explanation. Subjects with test abnormalities can be quickly referred to experts for a more detailed assessment [16,17].

- SPMSQ

The SPMSQ was proposed by Pfeiffer in 1975 and used to measure consciousness, memory, orientation, attention, thinking, and general knowledge to understand the current mental health status of the subject preliminarily [18]. One can be self-tested or take the test with the assistance of family members for preliminary dementia screening. Related studies found that the test results vary based on education level but do not change with age [19].

- MMSE

The MMSE is a cognitive function assessment form designed by Folstein et al. in 1975 and is used to assess overall cognitive function [20]. The assessment questions are related to orientation (time and place), language (reading, writing, naming, and comprehension), construction (visual drawing), attention and calculation (message confirmation and continuous subtraction of a specific number), and memory (short-term memory). There is no time limit; the maximum attainable score is 30 points. When the test score is 23–25 points, attention should be paid to the possibility of MCI [21].

- SLUMS

The SLUMS was published by Morley and Tumosa in 2002 and contains 11 items [22]. Subsequently, Zheng et al. (2012) revised it to eight cognitive functions: orientation, calculation, image recognition, animal naming, number sequence, memory, executive function, and responsiveness [23]. The test’s maximum attainable score is 30 points. If the subject has completed senior high school, a test score of 21–26 points indicates possible MCI. The SLUMS has higher sensitivity in subjects with a higher education level [24].

- CDR

The CDR was designed by Hughes et al. in 1982 to assess senile dementia of the Alzheimer’s type [25]. Subsequently, Morris (1993) added some scope based on the evaluation rules and developed the current clinical scale [26]. The scale items include cognitive and functional performance in six areas: memory, orientation, judgment and problem solving, community affairs, home and hobbies, and personal care. Related studies showed that the CDR has some reliability in predicting MCI; at a CDR of 0.5 points, 37.3% of patients progressed to AD [27].

- CASI

The CASI was published by Teng et al. in 1994 and integrated the Hasegawa Dementia Scale from Japan, MMSE, and DSM-III-R diagnostic criteria [28]. The scale items include ten items, attention, concentration, orientation, short-term memory, long-term memory, language abilities, visual construction, list-generating fluency, abstraction, and judgment. The total score is 100 points; different levels of education have different judgment scores for MCI.

The six neuropsychological tools sometimes are used in different settings: Mini-Cog or the SPMSQ for a brief and quick examination in outpatient clinics or for hospitalized geriatric patients and the MMSE and CDR in primary care for cognitive impairment screening. Additionally, each screening tool requires time for explanation and should be taken care of, even when using a paper and pencil. There may be resistance from the subject if he/she has to undergo many screening tests simultaneously for multiple evaluation information, resulting in execution difficulty [29]. It is difficult to perform multiple neuropsychological tests simultaneously on a doubted patient. However, screening for cognitive decline in the primary care setting with one or two of the neurological tests is important. Then, the MCI should be confirmed in a memory clinic or specialized consultancy. If multiple neurological tests can be combined into one test, the result will be useful diagnostic reference information for clinicians or clinic workers.

3D virtual reality is widely used due to advances in information technology. If the six neuropsychological MCI screening tests can be designed in one test with an entertaining scenario via a 3D virtual environment, middle-aged to older adults may be incentivized and drawn to participate in cognitive screening and subsequent continuous follow-ups, resulting in more timely cognition data for detecting cognitive decline. The objectives of this study are to use 3D virtual reality to create an entertaining test scenario integrating the Mini-Cog, SPMSQ, MMSE, SLUMS, CDR, and CASI for middle-aged to older adults, incentivizing and drawing them to participate in the test, and, furthermore, to employ fuzzy logic control (FLC) technology to develop a “MCI assessment system” for obtaining some pilot information for MCI assessment. When the subject finishes the test with the “MCI assessment system,” there are two results that will be presented. First, the testing results corresponding to the six traditional neuropsychological tools are presented by images. Second, a MCI assessment score is generated and shown in three grades (green for normal situations, yellow for doubted MCI, and red for confirmed MCI) to provide medical workers with an evaluation reference.

## 2. Materials and Methods

### 2.1. Development of MCI Assessment System

#### 2.1.1. Framework

**Figure 1:**
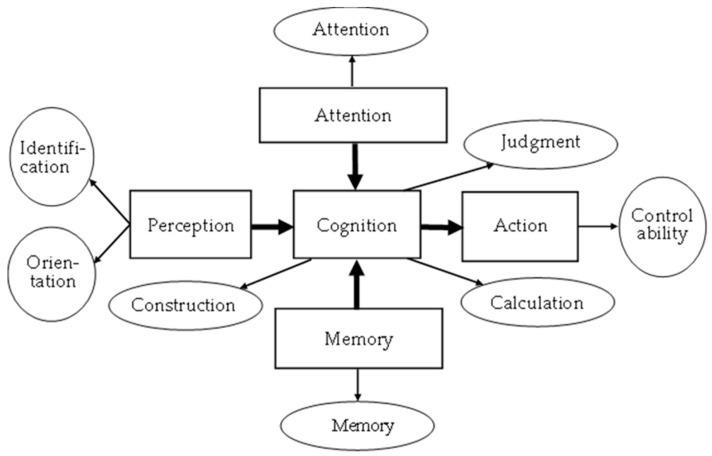
Relationship between eight major mild cognitive impairment tests (circle mark) and human information processing functions (square mark) [30].

**Figure 2:**
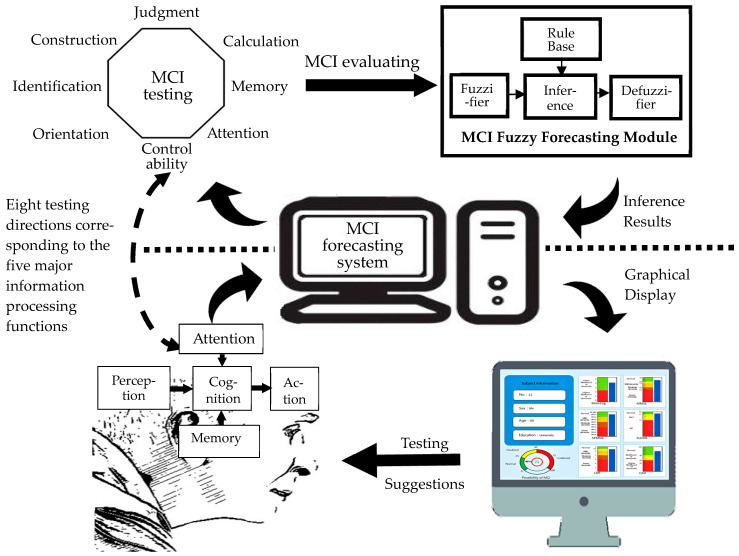
Framework of the mild cognitive impairment (MCI) assessment system.

Based on human information processing functions, eight major mild cognitive impairment tests were to be constructed, as shown in Figure 1. Figure 2 shows the framework of the MCI assessment system. This system applied virtual reality to construct a test environment. It collected test information from eight major dimensions for MCI evaluation. It then used FLC technology to develop the back-end system calculation platform to obtain an integrated assessment of the possibility of MCI in the subject. The MCI assessment results are displayed as MCI possibilities on the dashboard. In this way, the scenario-based image test proceeding in a virtual MCI testing center can allow middle-aged to elderly subjects to undergo testing while in a visiting mood, thereby reducing the worry and boredom associated with paper testing. The situational guidance of the virtual environment can let the subjects join the test at ease so that whether the subjects suffer from MCI and its severity will be assessed more precisely.

#### 2.1.2. Assessment Method

This study applied the concept of weight ratio based on the scoring methods, matching the scoring results of each item with the evaluation scores of the six traditional screening tools, thereby obtaining the scores of each screening tool (Table 1). Therefore, subjects can obtain assessment scores for six traditional screening tests by taking only one. With these different test dimensions, the subjects’ problematic items can be further examined in the future to determine which human information processing functions are affected. Based on eight major mild cognitive impairment tests, eleven assessment sub-items were formulated based on scenario concepts, ensuring the assessment method and content of the original assessment tools. Taking calculation as an example, most screening tools use stereotyped numbers for subjects to calculate, such as the SPMSQ (subtract 3 for a total of 5 times, starting from 20); MMSE (subtract 7 for a total of 5 times, starting from 100); CDR (subtract 3 for a total of 5 times, starting from 20); and CASI (subtract 3 for a total of 5 times, starting from 100). This may cause the subject to be impatient and develop psychological resistance, affecting the tools’ accuracy [29]. Additionally, we constructed a virtual fish tank and simulated fish swimming. The subject was invited to this test room and observed the fish swimming around naturally before answering questions on fish calculation relaxedly. This would increase subject acceptability.

#### 2.1.3. MCI Fuzzy Assessment Module

After establishing the assessment items in Table 1, each assessment item’s values were weighed to match the various assessment items to the original screening tool scores; however, we had to consider how to obtain the best evaluation value of MCI from the test results of the six traditional screening tools. Fuzzy logical control (FLC), based on fuzzy set theory, is a method that could be considered. Zadeh proposed fuzzy set theory in 1965 [31] and the research on FLC began in “Decision-Making in a Fuzzy Environment” published by Bellman and Zadeh in 1970 [32]. The core of FLC consists of a fuzzy rule base and a fuzzy inference engine (FIE). This study used FLC mode to construct the MCI assessment module (Figure 2). The top-right block of this figure shows the fuzzy assessment mechanism with a FLC core.

The scores of six traditional screening tools in the MCI fuzzy assessment mechanism were used as the FLC’s membership function and the rule base was used to generate fuzzy inference. The FIE assessed whether the subject was normal, suspected of MCI, or suspected of AD. First, the fuzzy inference semantic function for variable inputs, degree of membership, fuzzy reasoning, and output were defined. Each variable (six traditional screening tools) was defined as an input variable, including three membership functions (normal, mild, and MS (moderate and severe)). The output variable was a cognitive impairment (CI) estimate, including three membership functions (normal, doubted, and confirmed). Therefore, the rule base was developed with 729 rules, of which 7 belong to normal, 57 to MCI (doubted), as shown in Table 2, and 665 to MCI (confirmed).

This study used a 5-layer mechanism of fuzzification, fuzzy inference, and defuzzification to obtain a cognitive impairment estimate (CI). The various fuzzy layer variables are defined as follows: *i* represents the *i*th inference principle; *j* represents the *j*th membership function of the Mini-Cog variable (MC); *k* represents the *k*th membership function of the SPMSQ variable (SP); *m* represents the *m*th membership function of the MMSE variable (MM); *n* represents the *n*th membership function of the SLUMS variable (SL); *o* represents the *o*th membership function of the CDR variable (CD); and *p* represents the *p*th membership function of the CASI variable (CA).

First layer: In this layer, the input variables are used to calculate the memberships of the Mini-Cog(MC), SPMSQ(SP), MMSE(MM), SLUMS(SL), CDR(CD), and CASI(CA) and the fuzzy membership functions in graphical ways are a triangle and a trapezoidal.

$$O_{1,j}=\mu_{MCj}\left(s\right)j=1,2,3$$

$$O_{1,k}=\mu_{SPk}\left(t\right)k=1,2,3$$

$$O_{1,m}=\mu_{MMm}\left(u\right)m=1,2,3$$

$$O_{1,n}=\mu_{SLn}\left(v\right)n=1,2,3$$

$$O_{1,o}=\mu_{CDo}\left(w\right)o=1,2,3$$

(1) $$O_{1,p}=\mu_{CAp}\left(x\right)p=1,2,3$$

Second layer: This layer processes the operation of the fuzzy inference’s front step and the Min operator is employed in this study.

$$O_{2,i}=\omega_{i}=\mu_{MCj}\left(s\right)\mu_{SPk}\left(t\right)\mu_{MMm}\left(u\right)\mu_{SLn}\left(v\right)\mu_{CDo}\left(w\right)\mu_{CAp}\left(x\right)\;=\underset{j,k,m,n,o,p}{\mathrm{Min}}\left\{\mu_{MCj}\left(s\right),\mu_{SPk}\left(t\right),\mu_{MMm}\left(u\right),\mu_{SLn}\left(v\right),\mu_{CDo}\left(w\right),\mu_{CAp}\left(x\right)\right\}$$

(2) $${i=1\sim 729;j,k,m,n,o,p=1,2,3}_{}$$

The *ω_i_* represents the firing strength of the *i*th inference rule.

Third layer: This layer calculates the weight of the firing strength of the *i*th rule to the firing strength of all rules.

(3) $$O_{3,1}=\overset{-}{\omega_{i}}=\frac{\omega_{i}}{\sum_{i=1}^{729}\omega_{i}}\;i=1\sim 729$$

Fourth layer: This layer calculates the firing strength of 729 rules and is the corresponding output function.

(4) $$O_{4,1}=\overset{-}{\omega_{i}}\cdot f_{i}\;i=1\sim 729$$

Fifth layer: This layer sums the outputs of 729 rules, i.e., obtains the cognitive impairment estimate: *CI*.

(5) $$O_{5,1}=CI=\sum_{i=1}^{729}{\overset{-}{\omega }}_{i}\cdot f_{i}=\frac{\omega_{i}\cdot f_{i}}{\sum_{i=1}^{729}\omega_{i}\cdot f_{i}}\;i=1\sim 729$$

#### 2.1.4. System Scenario Design

To effectively develop the “MCI assessment system” and address issues related to hardcopy tests, there were four steps to develop the system: theoretical development, system analysis, system design, and system testing. In this study, in addition to using virtual reality (VR) to construct a scene image test environment and using navigation instructions to guide the subjects to join in the test, whether the subjects suffer from MCI and its severity can be assessed more precisely. Eleven test regions were based on eight major dimensions to be designed in a MCI testing center. The objects and frame were drawn by 3D MAX and combined to obtain a 3D model for paintings.

- System Startup

**Figure 3:**
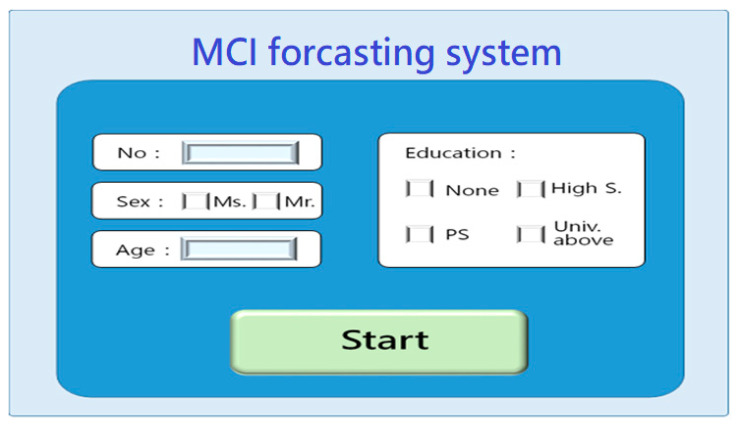
MCI foresting system-startup screen.

**Figure 4:**
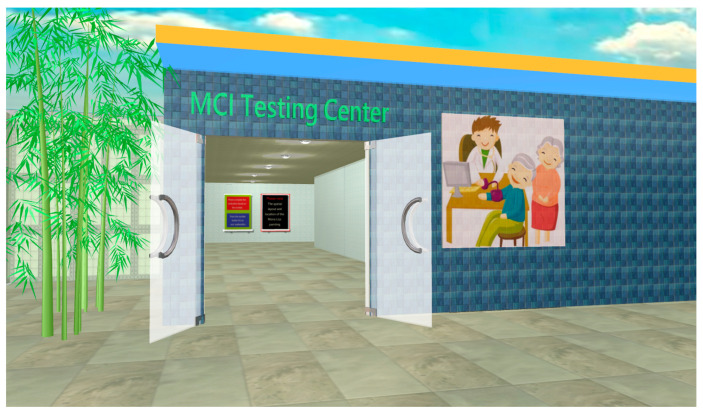
MCI testing center-entrance screen.

After the subject enters the MCI testing center, he/she first keys in personal general information (Figure 3) for the future comparative analysis of general information. After that, the MCI test center is activated and the subject goes into the center (Figure 4). The subject could use the keyboard or mouse to process the tests in the center.

- Orientation Test

**Figure 5:**
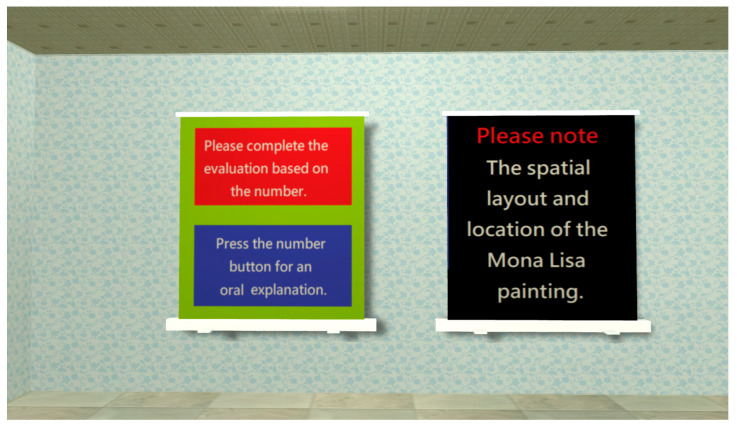
When entering the orientation test, an operation prompt will appear and remind the subjects to pay attention to the appearance of the Mona Lisa painting.

**Figure 6:**
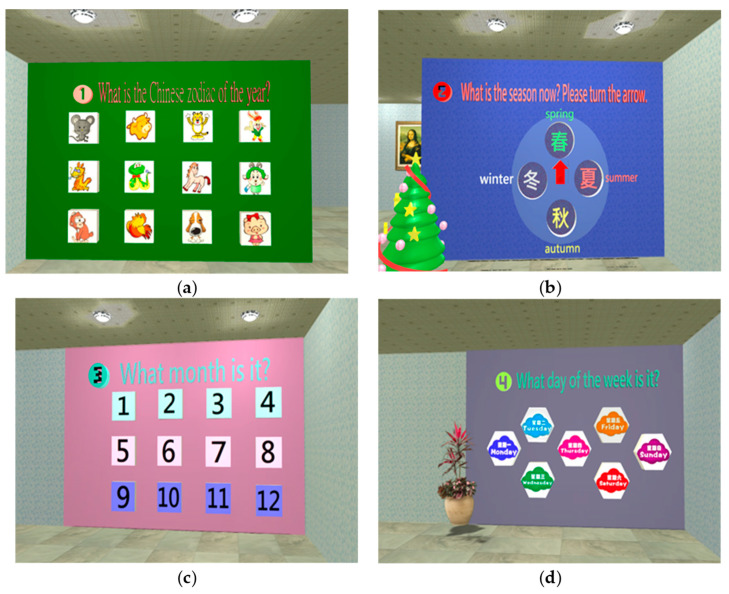
There are four types of orientation tests: (**a**) Chinese zodiac test: clicking on the Chinese zodiac sign of this year [30]; (**b**) Season test: rotating the arrow to confirm the current season; (**c**) Month test: clicking the current month; (**d**) Week test: clicking the day of the week today.

**Figure 7:**
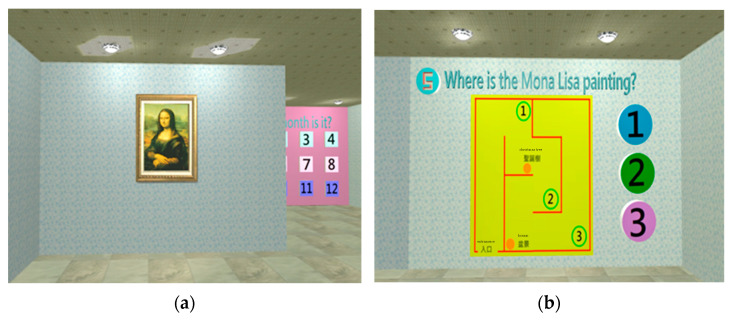
(**a**) When the “time” tests are underway, the Mona Lisa painting will appear; (**b**) the subject must click the location number of the Mona Lisa painting on the map.

The orientation test includes two dimensions (space and time); the ability to identify the place and time (i.e., where? and when?) would be evaluated. Figure 5 shows the operation notes displayed at the start of orientation test. Chinese zodiac, seasons, months, and weeks are used to assess the time ability of the subject (Figure 6); the location of the Mona Lisa painting is used to assess space abilities (Figure 7).

- Identification Test

**Figure 8:**
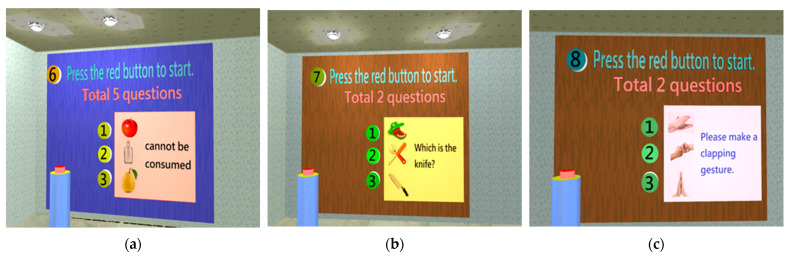
When entering the orientation test, three types of tests need to be completed: (**a**) Language expression test (total of five questions), e.g., clicking what cannot be eaten [30]; (**b**) Image recognition test (total of two questions), e.g., clicking the image of a knife; (**c**) Language comprehension test (total of two questions), e.g., selecting the action of clapping.

If the left temporal lobe is damaged, it will affect language expression and comprehension, thereby indirectly affecting memory [33]. The abilities of expression, recognition, and comprehension in language are tested in the identification test room to assess the identification abilities of the subject (Figure 8).

- Memory Test

**Figure 9:**
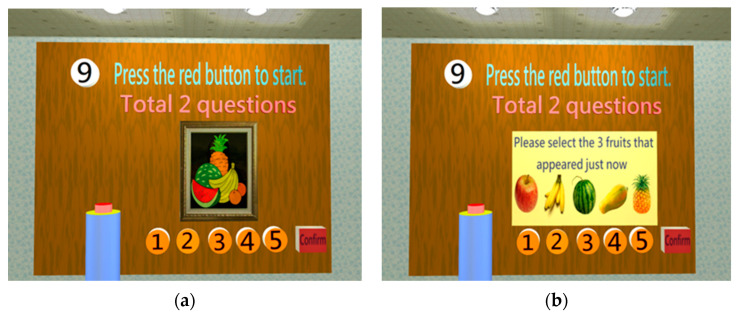
When entering the memory test, two questions need to be completed: object shape and image connection and object color and image connection: (**a**) Object shape and image connection: a fruit drawing appears for 5 s before disappearing; (**b**) a fruit list appears and the subject must select the fruit that had just appeared [30].

**Figure 10:**
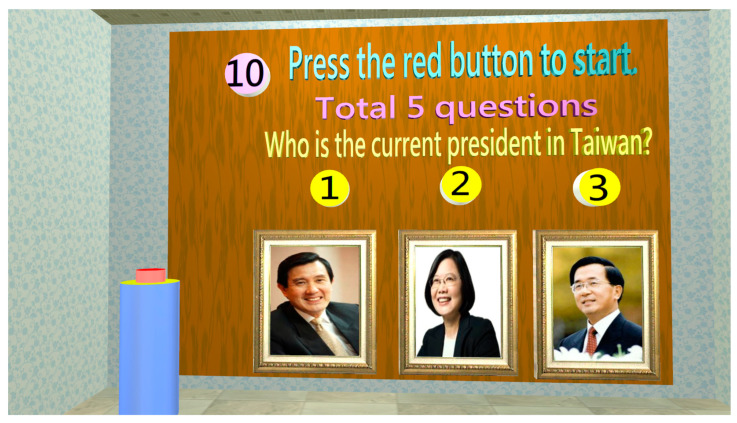
When the short-term memory tests are finished, the long-term memory tests will be started. There are five questions that need to be completed, such as selecting the current president of Taiwan.

Marquié et al. (2019) found that the ability of making visual connections would decline in short-term memory loss, such as those regarding object color and image. This is an early symptom of AD patients [34]. Therefore, the tests of the connection between object color and image and the connection between object shape and image could evaluate short-term memory (Figure 9). The test of long-term memory is mainly to evaluate memory loss for time sequence events. Figure 10 shows that a test for identifying the current president of Taiwan is designed to assess long-term memory.

- Attention Test

**Figure 11:**
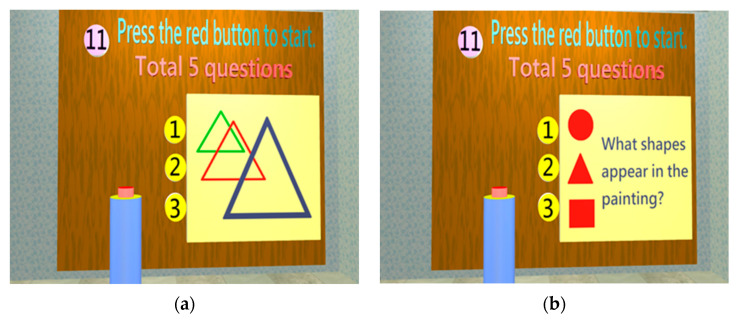
When entering the attention test room, five questions need to be completed: (**a**) A triangle pattern painting will appear for 5 s; (**b**) the subjects must choose the correct answer on the answer board for the pattern he or she saw.

Attention is the ability to perceive the external environment. The common causes of attention loss include dementia, loss of consciousness, irritability, depression, schizophrenia, or significant illness. Poor attention to a subject may suggest the presence of Alzheimer’s disease [35]. In the attention test room, the subject must pay attention to the randomly presented images and, according to the features of the images, choose the correct answer within a short time (Figure 11a,b).

- Construction Test

**Figure 12:**
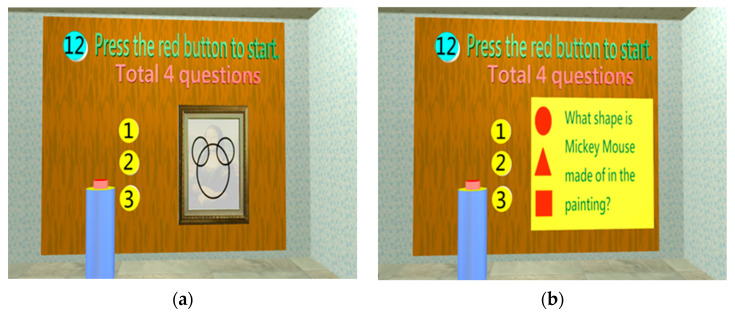
Four questions need to be completed when entering the construction test room: (**a**) A test of Mickey Mouse composition: a Mickey Mouse painting will appear for 5 s; (**b**) the subjects choose the correct answer on the answer board for the pattern he or she saw.

When a dementia patient develops progressive memory loss, his/her construction ability will be affected [36]. The objective of the construction test is to assess whether the subject can understand the causality of an event and has the ability to understand the composition of an event. In the construction room, the subject would be asked what shape makes up the image in the painting. The purpose is to test whether the subject can interpret the composition of the image (Figure 12a,b).

- Judgment Test

**Figure 13:**
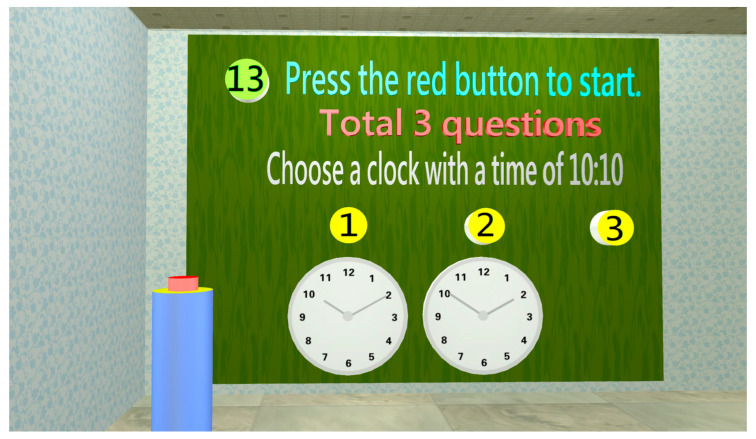
When entering the judgment test room, three questions need to be completed, such as judgment of a clock time display: the subject must chose the correct clock showing 10:10.

People with early dementia experience memory loss, followed by poor judgment [37]. In this study, (a) 10:10 judgment (Figure 13), (b) tools used by a physician, and (c) tools used by a teacher are designed for judgment assessment.

- Calculation Test

**Figure 14:**
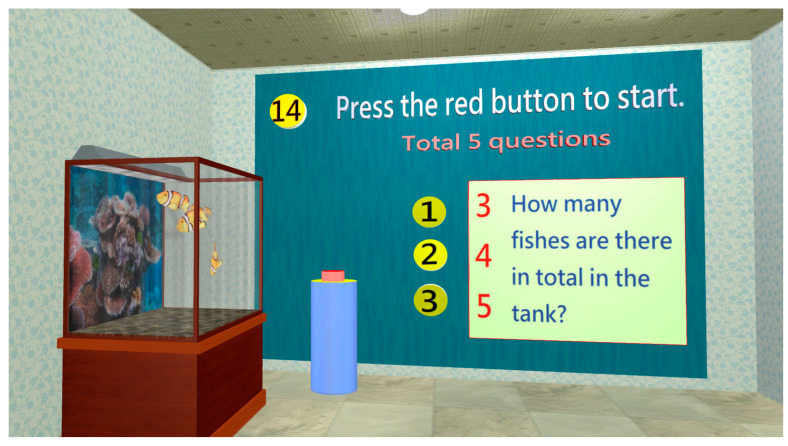
The subject enters the calculation assessment room and five questions need to be completed, such as calculating the number of fish.

During calculation, short-term memory is required in addition to logical thinking. When cognitive impairment occurs, memory decline will affect calculation; therefore, the worsening calculation may be auxiliary proof of cognitive impairment [38]. In the calculation room, the subject would observe fish in a fish tank (Figure 14) and count the number of fish.

- Control Ability Test

**Figure 15:**
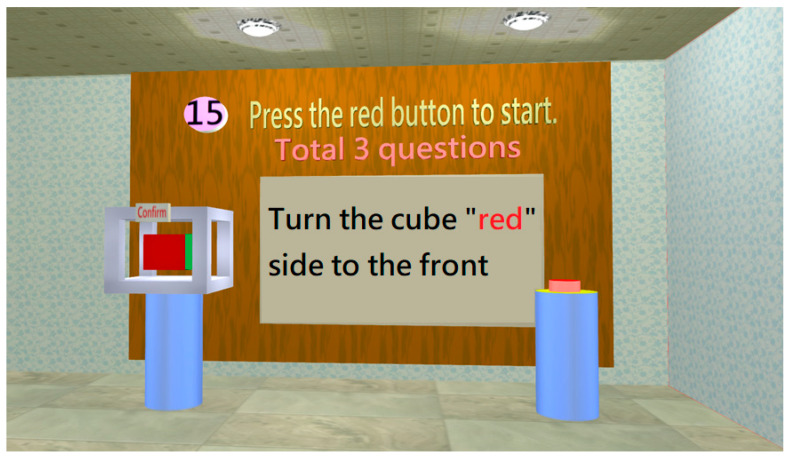
Three questions should be completed in the control ability test. When the subject enters the control ability assessment room, he/she will first see a display shelf with a cube. The subject must use the mouse to rotate the cube to the designated color (red) cube. The test is considered to have been failed if the reaction time exceeds 20 s.

Chung et al. found that control ability is auxiliary evidence of cognitive impairment. Control ability will also worsen when cognition is impaired [39]. In the control ability test room, the subject’s control ability is evaluated through the mouse control ability (Figure 15).

- Test Results Screen

**Figure 16:**
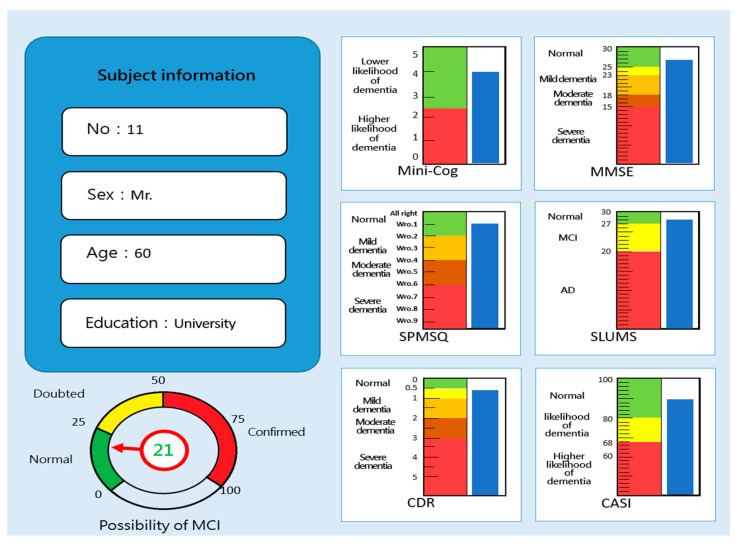
Results of the MCI assessment system [30].

Figure 16 shows the results of the MCI assessment by the eight major mild cognitive impairment tests. In the figure, the right image shows the corresponding scores of the six traditional screening test tools in which the scores undergo weighted conversion (blue rectangles show the corresponding scores) after the test. The evaluating grades of the six traditional screening tests are also displayed to facilitate identification. The various grades are presented in different colors, based on the evaluating criteria (e.g., green indicates normal and red indicates severe dementia). The lower left image shows the subject’s MCI assessment score, as calculated by fuzzy logic control. There are three grades in total: 0–25 points indicate an extremely low possibility of MCI and are shown by the green area; 26–50 points indicate doubted MCI and are shown by the yellow area; 51–100 points indicate confirmed MCI and are shown by the red area. Therefore, the higher the score, the more likely the subject is to suffer from MCI. The presentation of this assessment indicator can provide clinic workers with integrated evaluating scores, particularly when the test results of the Mini-Cog, SPMSQ, MMSE, SLUMS, CDR, and CAS are inconsistent.

### 2.2. Experimental Design

#### 2.2.1. Subjects

Generally, middle-aged and elderly people aged 50 to 65 are suspected to be susceptible to early MCI. In this study, 24 middle-aged and elderly people belonging to this age group were invited to participate in the MCI assessment test. All subjects were tested for visual acuity and had experience using computers in order to reduce individual differences.

#### 2.2.2. Experimental Procedure

A. Before testing, the operating instructions for the MCI assessment system were given to the subject and the subject was taught how to operate the system. The experiment was carried out after the subject was familiar with the system’s operation.

B. When the test started, the subject operated the personal computer or laptop by himself/herself and the assistant guided the subject to the screen entrance. After introducing the test items, the subject carried out various test operations based on instructions. During the test, the assistant would be at the side to assist. When the subject completed the test, he/she was required to complete a usability questionnaire to understand the system’s convenience. The test would be immediately stopped if the subject experienced discomfort during the test. In addition, one month after the test was completed, the subject would undergo testing with the six traditional screening tools: the Mini-Cog, SPMSQ, MMSE, SLUMS, CDR, and CASI. One test was performed each time, followed by an interval of 1 week before the next test was performed. During the test, the assistant would be at the subject’s side to assist in completing the hardcopy tests.

#### 2.2.3. Effectiveness Assessment

- Correlation Analysis

When the subjects completed the MCI assessment system test and six traditional screening tests, the MCI assessment system would generate corresponding scores for the six traditional screening tests. This study used Pearson’s correlation analysis on the corresponding scores of the six traditional screening tests by the MCI assessment system and the scores of the six traditional screening tests.

B. Usability Analysis

This study used the System Usability Scale (SUS) developed by Brooke in 1986 for the usability assessment of the MCI assessment system [40]. The SUS is widely used to rapidly detect the usability of product system interfaces, desktops, and website pages. Based on small sample study results by experts, the SUS can also be used for the quantitative analysis of small sample sizes (12 people). In order to facilitate an adequate understanding of SUS scores, Bangor et al. (2009) conducted a score perception study on the SUS in 2009 and divided SUS scores into five grades, namely, A: 90–100, B: 80–89, C: 70–79, D: 60–69, and F: 0–59 [41].

## 3. Results and Discussion

### 3.1. Correlation Analysis of the MCI Assessment System

Table 3 shows that the results of the corresponding scores generated by the MCI assessment system have a strong correlation with those of the traditional screening tests of the SPMSQ, Mini-Cog, MMSE, CASI, and SLUMS. In particular, the correlations with the MMSE and CASI are very high; but, the correlation with the CDR is moderate. The reason may be that the full score of the CDR test is only 3 points and the difference between each level is 0.5 points. When the MCI evaluation system calculates the CDR evaluation value, there may be large changes in the evaluation value due to slight differences in the responses of the subjects. Overall, the corresponding scores generated by the assessment system are consistent with some traditional screening tests. When doubts exist in one hardcopy test and other hardcopy tests are required, this may cause resistance in subjects, resulting in execution difficulty. Tests using our assessment system can be converted simultaneously to the traditional screen tests’ scores (Mini-Cog, SPMSQ, MMSE, SLUMS, and CASI) and correlate exceptionally well with them, significantly decreasing the difficulty of conducting multiple tests.

### 3.2. Effectiveness Analysis of the MCI Assessment System

Table 4 shows the comparative analysis of the “MCI assessment system” test and the hardcopy tests. Orange areas in the table show that the test met MCI criteria. The experiment results showed that the criteria for MCI symptoms were simultaneously met in the “MCI assessment system” test and the traditional screening tests for subjects No. 4, No. 10, and No. 23, showing that it is almost confirmed that these three subjects have MCI and may even have AD symptoms. These subjects were recommended to undergo further evaluation in a medical institution or undergo related medical measures. Subject No. 20 had suspected MCI results in the traditional Mini-Cog and CDR and the corresponding Mini-Cog and SLUMS results of this assessment system showed suspected MCI. This result showed that the subject may be close to the cutoff for MCI and further follow-up is required. It is recommended that the subject undergoes another test after 2–3 weeks to confirm whether the symptoms are present or goes to a medical institution to assess disease progression.

Table 4 also shows that most subjects have highly suspected MCI results for the SLUMS in the assessment system. This result showed that the assessment system might have higher sensitivity in converting the SLUMS scores, resulting in the MCI case. At this point, other test scores should be used as a basis for judgment. Generally, the screening analysis results found a high correlation between the MCI assessment system and traditional screening tests. The assessment system would also show similar results if multiple traditional screening tests showed that the subject had MCI or AD. If only a few traditional tests showed that the subject had MCI or AD, the assessment system would also show data for these corresponding tests but with some differences. When there are differences between the assessment system and traditional screening tests, the subject should be followed up with continuously. Overall, the results of this assessment system are reliable.

### 3.3. Effectiveness Analysis of the Fuzzy Measurement

**Figure 17:**
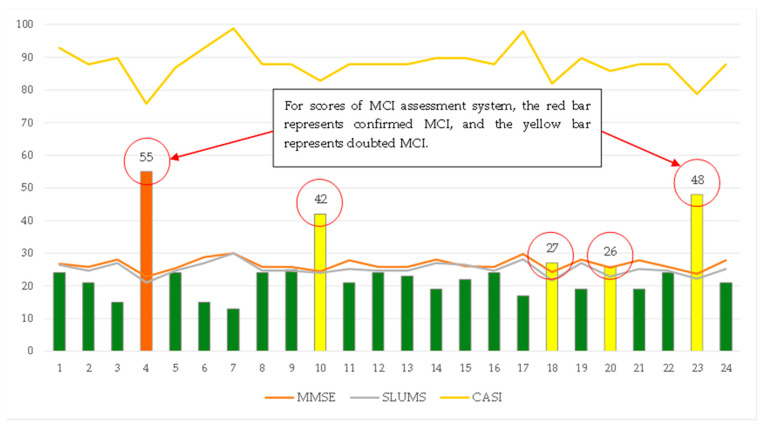
Distribution of the scores of the MCI assessment system test and hardcopy tests (MMSE, SLUMS, and CASI) [30].

In order to improve assessment when using the “MCI assessment system” test, fuzzy logic was used to construct a MCI fuzzy prediction module in this study. This module used the scores of six major assessment tools as membership functions for FLC (fuzzy logical control) and a rule base was used to generate fuzzy inference. The purpose of outputting results is to provide clinic workers with MCI reference values. Figure 17 shows the distribution of the scores of the MCI assessment system test and hardcopy tests (MMSE, SLUMS, and CASI). In particular, subject No. 20 showed that the test scores were inconsistent between the traditional screening tests: the traditional Mini-Cog and CDR showed suspected MCI results but the SPMSQ, MMSE, SLUMS, and CASI showed normal results. At this time, the evaluation score of the assessment system was 26 points, reaching the suspected MCI score. Therefore, it can be judged that the subject was a suspected MCI patient. In addition, subject No. 4 showed suspected MCI in all six traditional screening tests. The evaluation score of the assessment system was 55 points, which belonged to confirmed MCI patients. This result also proves the credibility of the MCI assessment system.

### 3.4. Usability of the MCI Assessment System

**Figure 18:**
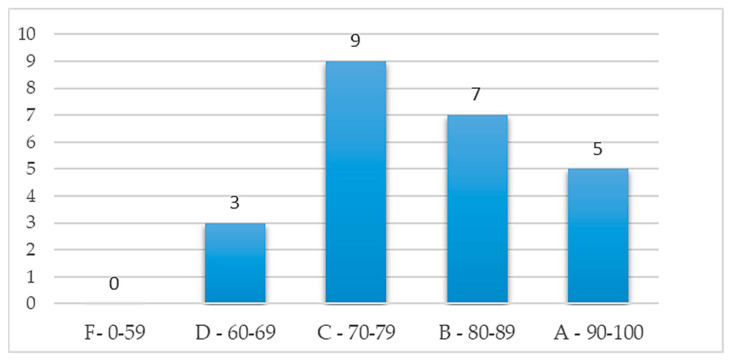
Distribution graph of usability grads.

**Figure 19:**
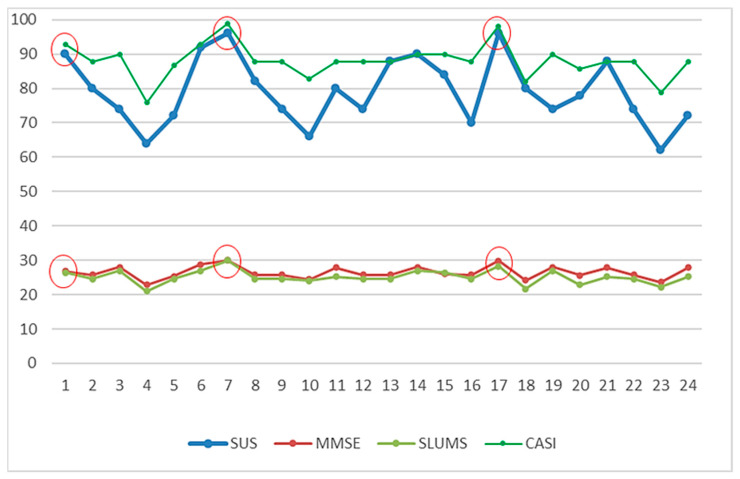
Correlation between usability use score and test scores. The circles shows that the higher the test score (better cognitive function), the greater the usability score.

Figure 18 shows the distribution of the system usability scale (SUS) evaluation scores for the MCI assessment system, which found that 87.5% were grade C (good to use) or above and 29.2% were grade B (extremely good to use) or above. This result shows that the assessment system has good feedback from the subjects. In addition, Figure 19 shows that the higher the test score (better cognitive function), the greater the usability score. It means that SUS scores could also indirectly reflect cognitive abnormalities. The subjects with cognition impairment would find it hard to use the assessment system because their orientation, control ability, or other abilities have declined. Further, this study obtained meaningful reactions from the subjects and experimental assistants in the operating process. With their responses, this study can understand their perspectives and these important responses will be the basis for improving the system. Table 5 shows the overall comments of the subjects and experimental assistants. The suggestion in “Demerits,” stating that “Testing paths should be marked more clearly,” presents a good opinion for system improvement. Additionally, the subject responded that the test involving “Turn the cube “red” side to the front” is hard to operate. In fact, this test was designed to evaluate the control ability. If the subject is a MCI case, he/she will find it hard to operate the “cube.” The other comments in “Demerits” show that practice before the test is important to subjects and the experiment assistants being familiar with the system and explaining it well is also important. Nevertheless, the other suggestions are mostly satisfactory. Finally, the results in terms of usability are also consistent with the report of Zucchella et al. (2014): 3D virtual games can potentially be new and effective tools in managing and treating cognitive impairments for screening pre-dementia conditions [42]. Therefore, the MCI assessment system has significant advantages by using an entertaining method for MCI screening, such as saving time and costs and providing multiple screening records. Additionally, using a dynamic scenario-change design will prevent subjects from being affected by prior learning effects effectively.

## 4. Conclusions

The world has become an aging society. With the rapid increase in middle-aged to older people, dementia has become an increasingly severe global public health problem. MCI is a condition characterized by a decline in brain function and is often an early sign of dementia. Currently, hardcopy questionnaires are mainly used for the evaluation of dementia signs in middle-aged to older people. However, there are some limitations of the current screening methods. The test process is often unsuccessful, resulting in differences in the test results. In addition, there may be resistance from the subject if he/she has to undergo many screening tests simultaneously, resulting in execution difficulty. This study used VR to create an entertaining test scenario integrating the Mini-Cog, SPMSQ, MMSE, SLUMS, CDR, and CASI for middle-aged to older adults, incentivizing and drawing them to participate in the test, and, furthermore, employed FLC to develop a “MCI assessment system.” The results showed that the corresponding scores generated by the MCI assessment system have a strong correlation with the traditional screening tests of the SPMSQ, Mini-Cog, MMSE, CASI, and SLUMS. In particular, the correlation with the MMSE and CASI is very high but the correlation with the CDR is moderate. Furthermore, when the test score of the subject is inconsistent with the corresponding tests, the reference score can provide an integrated judgment to reduce confusion and to achieve the objective of warning. In addition, concerning usability, the assessment system was positively received by subjects. If the MCI assessment system uses an entertaining method for dementia screening, middle-aged to older people may be incentivized and attracted to participate in cognitive screening and subsequent continuous follow-ups, thereby obtaining more timely cognition data for the identification of cognitive decline. Therefore, there are significant advantages to using an entertaining method for MCI screening, such as saving time and costs and multiple screening records decreasing psychological stress. Because the study focused on developing and piloting the MCI assessment system with VR and fuzzy technology, there are some limitations at this stage, such as smaller data sets and those over the age of 65 not being included. In the future, the advanced study will be considered with a sample of normal people and cognitively impaired individuals aged 50–65, 66–80, and 81+ in a cross-over design; more tests are needed to test the system’s diagnostic accuracy for MCI and dementia. Lastly, as the MCI assessment system needs to be performed on a computer, there will be hardware requirements if used for testing.

## Figures and Tables

**Table 1:** Evaluation scores of assessment items are assigned to six traditional screening tools [30].

Stage	Dimension	Sub-Item	Content	Mini-Cog	SPMSQ	MMSE	SLUMS	CDR	CASI
Perception	Orientation	1. Orientation	1. Temporal orientation: (1) Zodiac sign? (2) What season is it? (3) What month is it? (4) What day of the week is it? 2. Spatial orientation: where is the Mona Lisa painting?		Number of correct answers	2–10	0.6–3	1. 1 point: 2 or 3 wrong answers; 2 points: 4 or 5 wrong answers 2. 0.5 points: wrong answer	
	Identification	1. Language expression	1. cannot be consumed (images: apple, bottle, pear) 2. Closed_(images: hands, eyes, pants) 3. I want to ride in a/an (images: mobile phone, airplane, camera) 4. I to eat (words: want, see, drink) 5. I walk (words: home, recollection, family)			0.4 0.4 0.4 0.4 0.4	-	-	1 1 1 1 1
		2. Image recognition	1. Images (toys, knives, tools); select the knife 2. Images (television, fan, refrigerator); select the television				-	0.5 points: 1 or 2 wrong answers	2.5 2.5
		3. Language comprehension	1. Clapping; select an image (clapping, applause, bringing the palms together) 2. Walking; select an image (jumping, walking, squatting)			0.5 0.5	-	0.5 points: 1 or 2 wrong answers	1.5 1.5
Memory	Memory	1. Short-term memory	1. Images of banana, pineapple, watermelon, etc., will appear and the subject is required to select the banana, pineapple, and watermelon from the 5 objects for a total of 2 times. 2. 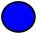 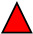 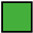 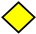 After memorizing, shape and color, matching is conducted twice.	1. 1 point for correctly answering all questions 2. 1 point for correctly answering all questions		1.5–6	1. 3–6 2. 3–6	0 points: 4 correct answers; 0.5 points: 3 correct answers; 1 point: 2 or fewer correct answers	5–30
		2. Long-term memory	1. Who is the current president of Taiwan? Photographs of Chen Shui-Bian, Ma Ying-Jeou, and Tsai Ing-Wen 2. Place of residence? Maps of Taiwan, Japan, and Hainan Island 3. How many minutes are there in 1 h? 4. How many months are there in 1 year? 5. In which direction does the sun set?		Number of correct answers	0.4–2		0 points: 4 correct answers; 0.5 points: 3 correct answers; 1 point: 2 or fewer correct answers	2–10
Attention	Attention	1. Attention	1. Random shapes: determine the shape as △, □, and ○; 5 consecutive times					1 point: 1 or more wrong answer	1.2–6
Cognition	Construction	1. Construction	1. How do you draw Mickey Mouse using △, □, and ○? 2. How do you construct 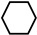 using △, □, and ○? 3. How do you construct 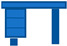 using △, □, and ○? 4. Label the 2 o’clock and 8 o’clock positions in the image (2 positions).	3 points: All correct answers 2 points: 1 wrong answer 1 point: 2 wrong answers 0 points: 3 or more wrong answers		0.2–1	2.4–12	0 points: 4 correct answers; 0.5 points: 3 correct answers; 1 point: 2 or fewer correct answers	3–15
	Judgment	1. Judgment	1. Determine if the time is 10:10 2. What do physicians use? (Images: scalpel, bottle, and wooden rod) 3. What do teachers use? (Images: pot, books, and hammer)					1 point: 2 wrong answers or more	2–6
	Calculation	1. Calculations	1. How many fish are there in total in the tank? 2. What is 3 + the previous answer? 3. What is 3 + the previous answer? 4. What is the previous answer + 3 twice? 5. You used 100 NTD to buy a 30 NTD apple and a 20 NTD orange. How much do you have left?		Number of correct answers	1 1 1 1 1	0.6 0.6 0.6 0.6 0.6	1 point: 3 wrong answers or more	1 1 1 1 1
Action	Control ability	1. Control ability	1. Rotate the cube red side to the front 2. Rotate the cube blue side to the front 3. Rotate the cube green side to the front			1–3		1 point: 2 wrong answers or more	1–3

**Table 2:** IF- MCI (normal and doubted) control rules base.

IF	MC	SP	MM	SL	CD	CA	CI	IF	MC	SP	MM	SL	CD	CA	CI
1	Nor	Nor	Nor	Nor	Nor	Nor	Nor	33	Mild	Nor	Nor	Mild	Nor	Mild	Dou
2	Mild	Nor	Nor	Nor	Nor	Nor	Nor	34	Nor	Mild	Mild	Nor	Mild	Nor	Dou
3	Nor	Mild	Nor	Nor	Nor	Nor	Nor	35	Nor	Mild	Mild	Nor	Nor	Mild	Dou
4	Nor	Nor	Mild	Nor	Nor	Nor	Nor	36	Nor	Mild	Nor	Mild	Mild	Nor	Dou
5	Nor	Nor	Nor	Mild	Nor	Nor	Nor	37	Nor	Mild	Nor	Mild	Nor	Mild	Dou
6	Nor	Nor	Nor	Nor	Mild	Nor	Nor	38	Nor	Mild	Nor	Nor	Mild	Mild	Dou
7	Nor	Nor	Nor	Nor	Nor	Mild	Nor	39	Nor	Nor	Mild	Mild	Mild	Nor	Dou
8	Mild	Mild	Nor	Nor	Nor	Nor	Dou	40	Nor	Nor	Mild	Mild	Nor	Mild	Dou
9	Mild	Nor	Mild	Nor	Nor	Nor	Dou	41	Nor	Nor	Mild	Nor	Mild	Mild	Dou
10	Mild	Nor	Nor	Mild	Nor	Nor	Dou	42	Nor	Nor	Nor	Mild	Mild	Mild	Dou
11	Mild	Nor	Nor	Nor	Mild	Nor	Dou	43	Mild	Mild	Mild	Mild	Nor	Nor	Dou
12	Mild	Nor	Nor	Nor	Nor	Mild	Dou	44	Mild	Mild	Mild	Nor	Mild	Nor	Dou
13	Nor	Mild	Mild	Nor	Nor	Nor	Dou	45	Mild	Mild	Mild	Nor	Nor	Mild	Dou
14	Nor	Mild	Nor	Mild	Nor	Nor	Dou	46	Mild	Mild	Nor	Mild	Mild	Nor	Dou
15	Nor	Mild	Nor	Nor	Mild	Nor	Dou	47	Mild	Mild	Nor	Mild	Nor	Mild	Dou
16	Nor	Mild	Nor	Nor	Nor	Mild	Dou	48	Mild	Mild	Nor	Nor	Mild	Mild	Dou
17	Nor	Nor	Mild	Nor	Nor	Mild	Dou	49	Mild	Nor	Mild	Mild	Mild	Nor	Dou
18	Nor	Nor	Mild	Nor	Mild	Nor	Dou	50	Mild	Nor	Mild	Mild	Nor	Mild	Dou
19	Nor	Nor	Mild	Mild	Nor	Nor	Dou	51	Mild	Nor	Mild	Nor	Mild	Mild	Dou
20	Nor	Nor	Mild	Mild	Nor	Nor	Dou	52	Mild	Nor	Nor	Mild	Mild	Mild	Dou
21	Nor	Nor	Nor	Mild	Nor	Mild	Dou	53	Nor	Mild	Mild	Mild	Mild	Nor	Dou
22	Nor	Nor	Nor	Nor	Mild	Mild	Dou	54	Nor	Mild	Mild	Mild	Nor	Mild	Dou
23	Mild	Mild	Mild	Nor	Nor	Nor	Dou	55	Nor	Mild	Mild	Nor	Mild	Mild	Dou
24	Mild	Mild	Nor	Mild	Nor	Nor	Dou	56	Nor	Mild	Nor	Mild	Mild	Mild	Dou
25	Mild	Mild	Nor	Nor	Mild	Nor	Dou	57	Nor	Nor	Mild	Mild	Mild	Mild	Dou
26	Mild	Mild	Nor	Nor	Nor	Mild	Dou	58	Mild	Mild	Mild	Mild	Mild	Nor	Dou
27	Mild	Nor	Mild	Mild	Nor	Nor	Dou	59	Mild	Mild	Mild	Mild	Nor	Mild	Dou
28	Mild	Nor	Mild	Mild	Nor	Nor	Dou	60	Mild	Mild	Mild	Nor	Mild	Mild	Dou
29	Mild	Nor	Mild	Nor	Nor	Mild	Dou	61	Mild	Mild	Nor	Mild	Mild	Mild	Dou
30	Mild	Nor	Nor	Mild	Mild	Nor	Dou	62	Mild	Nor	Mild	Mild	Mild	Mild	Dou
31	Mild	Nor	Nor	Mild	Nor	Mild	Dou	63	Nor	Mild	Mild	Mild	Mild	Mild	Dou
32	Mild	Nor	Nor	Mild	Nor	Mild	Dou	64	Mild	Mild	Mild	Mild	Mild	Mild	Dou

**Table 3:** Correlation analysis of test results of the traditional screen tests and the “MCI assessment system” test.

Test Method		The Corresponding Scores Generated by “MCI Assessment System” Test					
		Mini-Cog	SPMSQ	MMSE	SLUMS	CDR	CASI
Traditional screening tests	Mini-Cog	0.7894	-	-	-	-	-
	SPMSQ	-	0.8020	-	-	-	-
	MMSE	-	-	0.8875	-	-	-
	SLUMS	-	-	-	0.7715	-	-
	CDR	-	-	-	-	0.6892	-
	CASI	-	-	-	-	-	0.9141

**Table 4:** Comparison results between traditional screening tests and the “MCI assessment system” test.

No	Traditional Screening Tests						The Corresponding Scores Generated by the “MCI Assessment System” Test					
	Mini-Cog	SPMSQ	MMSE	SLUMS	CDR	CASI	Mini-Cog	SPMSQ	MMSE	SLUMS	CDR	CASI
1												
2												
3												
4												
5												
6												
7												
8												
9												
10												
11												
12												
13												
14												
15												
16												
17												
18												
19												
20												
21												
22												
23												
24												

Note: Orange areas in the table show that the test met MCI criteria.

**Table 5:** Overall comments of subjects and experimental assistants.

	Merits	Demerits
Subjects	It was funny. I thought that I would be willing to use this system next time. After practice, I thought the system was easy to use. Click the button directly for the answer; I thought the system was simple. The operation is very simple, just click the button. I felt very confident using the system after the test. The assistant explained it clearly and I was able to operate it by myself soon after.	I would need the support of a technical person in the beginning. I thought the test “Turn the cube “red” side to the front” was hard to operate. Testing paths should be marked more clearly.
Experimental Assistants	The experiments are clear and easy to explain. Most of the subjects were able to operate by themselves after explanation and practice.	During the practice, some subjects were less confident and needed more explanations.

## Data Availability

The data presented in this study are available on request from the corresponding author. The data are not publicly available due to privacy reasons.
